# Supplementary material for: Reconciling Mining with the Conservation of Cave Biodiversity: A Quantitative Baseline to Help Establish Conservation Priorities
Source: PLoS One. 2016 Dec 20;11(12):e0168348. doi: 10.1371/journal.pone.0168348 (PMC5173368; doi:10.1371/journal.pone.0168348)
Supplement: S1 Dataset — (ZIP) [file pone.0168348.s002.zip › Taxa/Serra Norte/SN_2013/N4WS/Lista N4WS-07.pdf]

CAVIDADE N4WS-0007

Classe	Ordem	Fam/Outros	Gên/Outros	Espécie	S-U	U-S	Zon.
Arachnida	Acari	(Parasitengonina)		sp.2		X	E
Arachnida	Acari	(Parasitengonina)		sp.3		X	P
Arachnida	Acari	(Uropodina)		sp.2	X		E
Arachnida	Acari	Anystidae	*Erythracarus*	*nasutus*		X	E
Arachnida	Acari	Macronyssidae		sp.1	X		E
Arachnida	Acari	Prostigmata		sp.1	X		P
Arachnida	Acari	Rhagidiidae		sp.1		X	E
Arachnida	Amblypygi	Phrynidae	*Heterophrynus*	*longicornis*	2	1	E/P
Arachnida	Araneae	Araneidae	*Alpaida*	*septemmammata*	X		E/P
Arachnida	Araneae	Araneidae	*Mangora*	sp.2	1		E
Arachnida	Araneae	Corinnidae		jovem	1		E
Arachnida	Araneae	Ctenidae		jovem	1		E
Arachnida	Araneae	Ochyroceratidae	*Speocera*	sp.1	X	X	E/P
Arachnida	Araneae	Ochyroceratidae	*Theotima*	sp.1		X	E
Arachnida	Araneae	Pholcidae	*Mesabolivar*	*aurantiacus*	X		P
Arachnida	Araneae	Pholcidae	Ninetinae	sp.1		X	E
Arachnida	Araneae	Salticidae		jovem	X		E
Arachnida	Araneae	Scytodidae	*Scytodes*	*globula*	5	5	E/P
Arachnida	Araneae	Theraphosidae		jovem	8		E
Arachnida	Araneae	Theridiosomatidae	*Plato*	sp.1	X	X	E/P
Arachnida	Opiliones	Escadabiidae		jovem	X		E
Arachnida	Opiliones	Sclerosomatidae	*Prionostema*	sp.1	X	X	E
Arachnida	Palpigradi	Eukoeneniidae	*Eukoenenia*	sp.1	X		P
Arachnida	Pseudoscorpiones	Chernetidae	*Spelaeochernes*	sp.1	X	X	E/P
Arachnida	Pseudoscorpiones	Chthoniidae	*Pseudochthonius*	sp.1		X	E/P
Chilopoda	Scolopendromorpha	Cryptopidae	*Cryptops*	sp.1		1	E
Diplopoda	Polydesmida	Pyrgodesmidae		sp.1		X	E/P
Entognatha	Collembola	Isotomidae		sp.1	X		P
Entognatha	Collembola	Paronellidae		sp.1	X	X	E
Entognatha	Collembola	Symphypleona		sp.1	X	X	E/P
Insecta	Archaeognatha	Meinertellidae		sp.1		X	E
Insecta	Blattodea	Blattidae		jovem		1	E
Insecta	Coleoptera	Lampyridae		jovem		X	E
Insecta	Coleoptera	Leiodidae		sp.3	X		E
Insecta	Coleoptera	Scydmaenidae		sp.13		X	E
Insecta	Coleoptera	Staphylinidae		sp.69		X	P
Insecta	Diptera	Ceratopogonidae		sp.	X		E
Insecta	Diptera	Chironomidae		sp.	X		E
Insecta	Diptera	Culicidae	*Culex*	sp.	X		E/P
Insecta	Diptera	Dolichopodidae		sp.	X		E
Insecta	Diptera	Ephydridae		sp.	X		E
Insecta	Diptera	Phoridae	Phorinae	sp.	X		E
Insecta	Diptera	Psychodidae	*Sciopemyia*	*sordellii*	X	X	E/P
Insecta	Diptera	Stratiomyidae		jovem	X		E
Insecta	Diptera	Tipulidae	Tipulinae	sp.	X		E/P
Insecta	Heteroptera	Reduviidae	Reduviinae	jovem	1		E
Insecta	Hymenoptera	Diapriidae		sp.2	X		P
Insecta	Hymenoptera	Formicidae	*Hypoponera*	sp.1		X	P
Insecta	Hymenoptera	Formicidae	*Pachycondyla*	*striata*	9	6	E/P

Insecta	Isoptera	Termitidae	*Nasutitermes*	sp.	X		E/P
Insecta	Lepidoptera	Noctuoidea		sp.1	1		E
Insecta	Lepidoptera			jovem		X	P
Insecta	Orthoptera	Phalangopsidae	*Paraclodes*	sp.	1	6	E
Insecta	Orthoptera	Phalangopsidae	*Phalangopsis*	sp.	22	3	E/P
Insecta	Psocoptera	Psocomorpha		jovem		X	E
Insecta	Thysanura	Nicoletiidae		sp.1	X		E
Gastropoda	Pulmonata	Subulinidae	*Lamellaxis*	sp.		X	E
Malacostraca	Isopoda	Philosciidae		sp.1	X		P
Malacostraca	Isopoda	Philosciidae		sp.2	X	X	E
Amphibia	Anura	Strabomantidae	*Pristimantis*	cf. *fenestratus*	1		E
Mammalia	Chiroptera	Emballonuridae	*Peropteryx*	sp.	7	13	E/P
Mammalia	Chiroptera	Furipteridae	*Furipterus*	*horrens*		3	P
